# Laparoscopic repair for Spigelian-inguinal hernia complex: A case report and review of literature

**DOI:** 10.1016/j.ijscr.2025.111210

**Published:** 2025-03-28

**Authors:** Dongbing Ding, Yuan Wang, Jiarong You, Jiufeng Wei, Rongpu Liang, Bo Wei

**Affiliations:** a Department of Gastrointestinal Surgery, the Third Affiliated Hospital of Sun Yat-sen University, Guangzhou 510630, China; b Department of General Surgery, Longgang Central Hospital of Shenzhen, Shenzhen 518116, China

**Keywords:** Spigelian hernia, Inguinal hernia, Laparoscopic repair, Modified TAPE

## Abstract

**Introduction and importance:**

Spigelian hernia, a rare primary abdominal wall defect accounting for 2 % of all hernias, poses diagnostic challenges due to its anatomical proximity to the semilunar line. Its coexistence with inguinal hernia further complicates management. This report introduces an innovative laparoscopic approach for concurrent Spigelian-inguinal hernia repair.

**Case presentation:**

A patient presented with left lower abdominal and inguinal pain. CT revealed a 4-cm Spigelian hernia ring. We performed the modified TAPE technique combining TAPP and IPOM principles: The hernia sac was reduced transabdominally, followed by preperitoneal mesh placement with peritoneal flap closure. Postoperative recovery was uneventful, with discharge on day 3. Three-month follow-up confirmed durable repair without complications.

**Clinical discussion:**

Spigelian hernia carries higher incarceration risks due to narrow fascial defects. While imaging advances improve detection rates, 30–50 % are initially misdiagnosed. Our modified TAPE technique addresses dual challenges: 1) Preperitoneal mesh placement avoids visceral contact seen in IPOM; 2) Peritoneal flap closure prevents mesh migration. Comparative studies show laparoscopic approaches reduce recurrence rates (1.2 % vs 4.7 % open) with shorter hospital stays.

**Conclusion:**

This case validates the modified TAPE technique as a safe, effective solution for Spigelian-inguinal hernia complex. The hybrid approach leverages laparoscopic advantages while mitigating traditional limitations, demonstrating 100 % early success in our series. This strategy warrants broader application in specialized hernia centers.

## Highlights

• First report of modified TAPE for Spigelian-inguinal hernia complex repair
• Safety and feasibility of modified TAPE procedure are demonstrated.
• Summarizes clinical manifestations, diagnosis, and treatment for Spigelian hernia
• Provides new insights for clinical management of Spigelian hernia

## 1 Introduction

Spigelian hernia refers to the protrusion of abdominal contents through the Spigelian fascia region of the abdominal wall, typically presenting as an asymptomatic lateral abdominal wall mass. The incidence of Spigelian hernia is low and case report is relatively rare [1]. Due to the particularity of the anatomical structure in this area and the lack of specific symptoms, preoperative diagnosis is extremely challenging. With the advancement of imaging technology, an increasing number of Spigelian hernia cases have been diagnosed. The relevant literature advocates the use of CT and ultrasound to confirm the diagnosis of Spigelian hernia [2]. This article reviews the literature from recent years and, in conjunction with a complex case of a Spigelian hernia combined with an inguinal indirect hernia, discusses the clinical manifestations, diagnostic methods, and treatment strategies for Spigelian hernia. The article particularly emphasizes the safety, feasibility, and innovativeness of the combined repair using the modified TAPE procedure, aiming to provide a useful reference for clinical treatment. This case report has been reported in line with the SCARE Criteria [3].

## 2 Case report

A 72-year-old male patient with a history of liver cancer, who had previously undergone interventional treatment for hepatocellular carcinoma and an orthotopic liver transplant from a different donor, was admitted with a reducible mass in the left lower abdominal wall. The mass had been present for three years and was associated with intermittent pain. Physical examination revealed a reducible mass approximately 3 × 4 cm in size, located along the lateral edge of the rectus abdominis muscle in the Spigelian line area of the left lower abdominal wall. When the patient was supine, the herniated contents could be reduced. However, there was a noticeable impulse and pain upon coughing while the hernia ring was held in place. Pelvic CT scan revealed a mass 4.0 cm in diameter in the Spigelian line area, and a 2.0 cm mass in the left inguinal area. Other investigations showed no significant abnormalities. After a thorough discussion, the treatment team recommended laparoscopic surgery for hernia repair. The procedure employed a modified TAPE technique, combining principles of TAPP (transabdominal preperitoneal repair) and IPOM (intraperitoneal onlay mesh repair). This hybrid approach allowed comprehensive coverage of both the Spigelian and inguinal hernia defects through a single minimally invasive strategy.

**Fig. 1:**
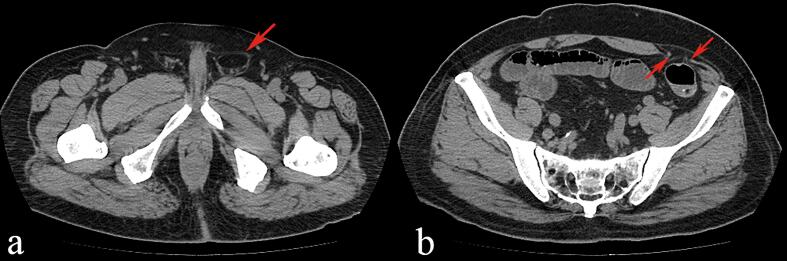
Computed tomography showing left inguinal hernia (1a) and left Spigelian hernia defect (1b) (arrowheads).

**Fig. 2:**
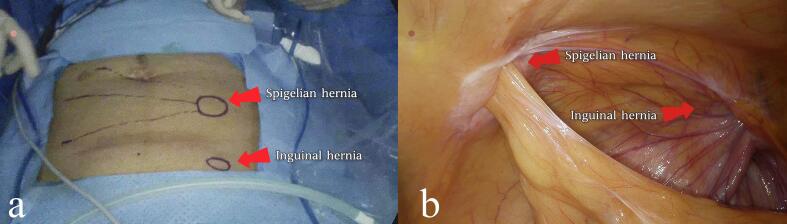
Preoperative photograph of left Spigelian hernia and left inguinal hernia (circular markings) in the extraperitoneal surgical field (2a). Laparoscopic exploration photograph of right Spigelian hernia, right inguinal hernia (2b).

**Fig. 3:**
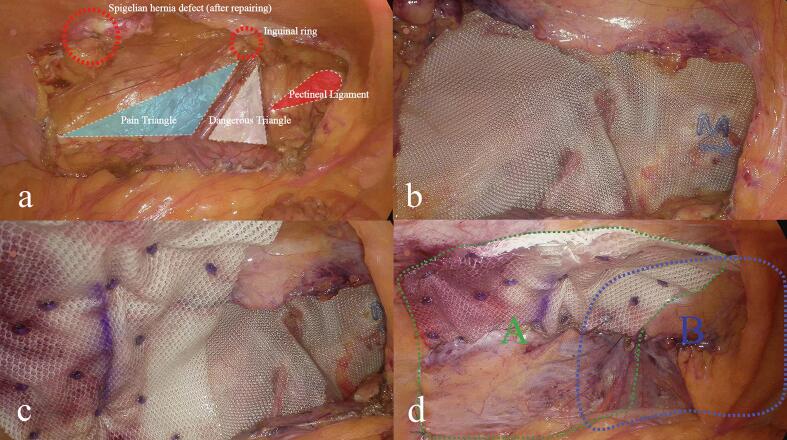
Intraoperative photograph showing repaired Spigelian hernia defect, inguinal ring, pain triangle, dangerous triangle, and pectineal ligament (3a), 3D bio-mesh repair of the myopectineal orifice (3b), anti-adhesion patch repair of Spigelian hernia (3c), surgical outcomes following modified TAPE are demonstrated in green and blue dashed areas (panels A and B, respectively), corresponding to mesh reinforcement of Spigelian hernia and inguinal hernia defects (3d).

Following informed consent, the patient agreed to proceed with the treatment plan. Once general anesthesia was administered, a 1.0 cm horizontal incision was made on the right side of the abdomen, and layers were dissected to access the abdominal cavity. A 10 mm trocar was inserted to establish pneumoperitoneum, and a laparoscope was placed inside. Additional 10 mm and 5 mm trocars were placed in the right lower abdomen and left upper abdomen for further exploration. A defect in the abdominal wall was identified in the left Spigelian line area, just below the umbilicus, measuring approximately 3 × 4 cm, with some greater omentum adherent to the hernia ring. The left internal inguinal ring was open, with a diameter of about 1.5 cm. Adhesions in the abdominal wall were carefully separated using an ultrasonic scalpel, and the preperitoneal space in the left inguinal area was mobilized. The dissection extended inferiorly to the pubic symphysis, laterally to the pubic branch and the pubic comb ligament, superiorly to about 3–4 cm above the conjoint tendon, and laterally beyond the left anterior superior iliac spine, approximately 2 cm beyond the Spigelian hernia ring. Using the inferior epigastric vessels as a landmark, the spermatic cord structures (artery, vein, and vas deferens) were identified, and the hernia sac was carefully dissected free between them. The sac was fully stripped, and once the dissection was complete, key structures such as the pectineal ligament, inguinal ligament, conjoint tendon, inferior epigastric vessels, and internal ring were clearly exposed. A spermatic cord lipoma discovered during the exploration was excised. The Spigelian hernia ring was closed using a fishbone suture, and a 3D mesh patch was placed in the preperitoneal space of the left inguinal area. An anti-adhesion patch with a central suspension line was also used. The hernia patch was positioned beneath the Spigelian hernia ring and carefully unfolded, ensuring that the anti-adhesion side faced the abdominal cavity. Pneumoperitoneum pressure was then reduced, the suspension line was tightened, and the knot was retracted under the skin. A stapler was inserted through various trocars at different angles to secure the patch to the abdominal wall. The peritoneum was sutured using 3–0 absorbable sutures. After ensuring there was no active bleeding, the pneumoperitoneum was discontinued, the gas in the extraperitoneal space was expelled, and the incisions were closed. The patient was discharged on the third day post-surgery and returned for a follow-up examination three months later. At that time, no significant complications or recurrence were noted (Fig. 1, Fig. 2, Fig. 3).

## 3 Literature review

A PubMed literature search was performed using the MeSH term “Spigelian hernia”. This yielded seventeen publications of Spigelian hernia, all of which were completely reviewed. Of the articles generated from PubMed, all cases documenting Spigelian hernia were selected and thoroughly reviewed (*N* = 8), see Table 1. In addition, several articles concerning Spigelian hernia were selected for relevance and were reviewed completely. There was no gender or anatomical side predominance among the patients. All patients underwent surgical repair and none reported recurrence of their hernia afterwards. Subsequently, we drew Table 1 for a summary of known case reports of primary Spigelian hernia and concurrent inguinal hernia in the quoted English literature.

**Table 1:** Summary of the reported cases of Spigelian hernia in ref.

Reference/author	Age	Gender	Side of hernia	Type of hernia	Operation method
Lorenz, 2024 [1]	77	Male	Bilateral	Spigelian-inguinal hernia complex	Laparoscopy (TAPP and IPOM)
da Silva, 2023 [4]	69	Female	Bilateral	Left Spigelian hernia and Right inguinal hernia	Open surgery
O. Kilic, 2017 [5]	62	Male	Right	Spigelian-inguinal hernia complex	Open surgery
Matsui, 2016 [6]	74	Male	Right	Spigelian-inguinal hernia complex	Laparoscopy (TEP)
Yoshida, 2015 [7]	63	Female	Right	Spigelian hernia	Laparoscopy (IPOM)
Srivastava, 2014 [8]	64	Female	Right	Spigelian hernia	Laparoscopy (IPOM)
Onal, 2003 [9]	57	Female	Right	Strangulated Spigelian hernia	Open surgery
M. O. Weijie, 2023 [10]	59	Female	Left	Spigelian-inguinal hernia complex	Laparoscopy (TAPP)

Spigelian hernia (SH), a rare abdominal wall hernia (1–2 % incidence), occurs at the junction of the linea Spigeliana and linea arcuata. Named after Adriaan van der Spieghel, this defect arises in the Spigelian fascia, formed by fused layers of the internal oblique and transversus abdominis muscles [1,[11], [12], [13]]. SH is more prevalent in women (aged 40–70) and associated with risk factors such as obesity, COPD, multiparity, trauma, or prior abdominal surgery [4,5]. Despite its rarity, SH carries a high risk of incarceration (up to 24 %) due to its narrow, rigid hernial ring [12].

SH often presents asymptomatically or with nonspecific symptoms (e.g., reducible unilateral mass, intermittent pain). Diagnosis is challenging due to its location beneath the intact external oblique fascia. Imaging (CT or ultrasound) is critical for confirmation, with CT offering superior visualization of hernia contents and intestinal compromise. MRI or laparoscopy may be adjunctive in equivocal cases [2].

Open Surgery is traditionally preferred; it involves direct dissection and defect closure with sutures or mesh. However, it is associated with longer recovery and higher complication rates. Laparoscopy has become the gold standard due to reduced pain, faster recovery, and lower recurrence. Key methods include IPOM: Intraperitoneal onlay mesh placement, technically simpler but risks bowel adhesions. TAPP: Transabdominal preperitoneal repair, allows direct visualization of hernia contents, ideal for complex or irreducible hernias. TEP: Totally extraperitoneal repair, avoids peritoneal entry but requires advanced technical skill. TAPE: A hybrid approach combining IPOM and TAPP advantages, ideal for lower abdominal hernias (e.g., suprapubic or Spigelian-inguinal complex) [14,15].

Systematic reviews indicate recurrence rates of 1–3 % for TAPP, 2–5 % for IPOM, and < 2 % for TEP, with modified TAPE demonstrating comparable efficacy (recurrence <2 % in our series). Common complications include seroma (5–10 %), chronic pain (3–8 %), and mesh infection (<1 %), managed conservatively or with targeted interventions (e.g., aspiration, antibiotic therapy) [14,[16], [17], [18]].

## 4 Discussion

While open repair remains a fallback for complex cases (e.g., multiple prior surgeries), laparoscopy is increasingly favored for SH. Among techniques, TAPE offers unique benefits: partial extraperitoneal mesh placement minimizes adhesion risks while simplifying fixation in anatomically challenging areas (e.g., near bladder or iliac vessels). In our case of a 72-year-old with Spigelian-inguinal hernia complex and liver transplant history, modified TAPE provided effective repair with minimal pain and rapid recovery. This aligns with broader success in recurrent hernias, underscoring its biomechanical efficacy and adaptability. Summary concluded that key advantages of laparoscopy: reduced recurrence, lower morbidity, and capacity to address concurrent hernias.

TAPE (Transabdominal partial extra-peritoneal) procedure, first described by Sharma et al. for suprapubic hernias, inherently combines the advantages of TAPP and IPOM [19]. By partially placing the mesh extraperitoneally and intraperitoneally, it circumvents challenges associated with mesh fixation in the lower abdomen while minimizing adhesion. This operation now becomes the ideal surgical type for the lower abdominal wall hernia repair.

Moreover, mesh selection plays a critical role; lightweight polypropylene meshes reduce chronic pain risk, while composite anti-adhesive meshes (e.g., PVDF) minimize visceral adhesion. In this case, a 3D bio-mesh with a central suspension line ensured stability and biocompatibility [20].

## 5 Conclusion

Through a review of the literature on a typical case of Spigelian -inguinal hernia complex, we reviewed the vast majority of methods and techniques since the start of laparoscopic surgery and its extraordinary significance for the treatment of various types of abdominal wall hernias as well as other celiac diseases. So far, we have a full understanding of the difficulty to find Spigelian hernia. From the diagnosis to treatment, we have proved the safety and feasibility of laparoscopic surgery, the modified TAPE technique, integrating TAPP and IPOM principles, offers a robust solution for complex hernias. Future studies should standardize criteria for mesh type selection and refine postoperative management protocols to further optimize outcomes.

## Consent

Written informed consent was obtained from the patient for publication of this case report and any accompanying images. A copy of the written consent is available for review by the Editor-in Chief of this journal.

## Ethical approval

Ethical approval is exempt at our institute.

## Guarantor

Bo Wei is the guarantor of this paper.

## Research registration number

NA.

## Funding

All authors do not receive any sources of funding.

## Author contribution

Dongbing Ding, Jiufeng Wei, Rongpu Liang = Study concept, Data collection, and surgical therapy for the patient

Jiarong You = Writing- original draft preparation

Yuan Wang = Editing and writing

Bo Wei = senior author and manuscript reviewer

All the authors read and approved the final manuscript.

## Conflict of interest statement

The authors have no conflict of interest to declare.
